# Quantitative proteomics revealed modulation of macrophages by *MetQ* gene of *Streptococcus suis* serotype 2

**DOI:** 10.1186/s13568-020-01131-2

**Published:** 2020-10-30

**Authors:** Xiaomeng Pei, Junchi Liu, Mingxing Liu, Hong Zhou, Xiaomin Wang, Hongjie Fan

**Affiliations:** 1 MOE Joint International Research Laboratory of Animal Health and Food Safety, College of Veterinary Medicine, Nanjing Agricultural University, Nanjing, China; 2 Department of Microbiology, Zunyi Medical University, No.6 West Xuefu Road, Xinpu District, Zunyi, China; 3 Jiangsu Co-innovation Center for Prevention and Control of Important Animal Infectious Diseases and Zoonoses, Yangzhou, China

**Keywords:** *Streptococcus suis* serotype 2, Quantitative proteomics, *MetQ*, Antiphagocytosis, Macrophages

## Abstract

*Streptococcus suis* serotype 2 (SS2) is a serious zoonotic pathogen; it can lead to symptoms of streptococcal toxic shock syndrome (STSS) in humans and sepsis in pigs, and poses a great threat to public health. The SS2 *MetQ* gene deletion strain has attenuated antiphagocytosis, although the mechanism of antiphagocytosis and pathogenesis of *MetQ* in SS2 has remained unclear. In this study, stable isotope labeling by amino acids in cell culture (SILAC) based liquid chromatography–mass spectrometry (LC–MS) and subsequent bioinformatics analysis was used to determine differentially expressed proteins of RAW264.7 cells infected with △*MetQ* and ZY05719. Proteomic results were verified by quantitative real-time polymerase chain reaction (qRT-PCR) and Western blotting for selected proteins. Further research was focused mainly on immune system processes related to downregulated proteins, such as Src and Ccl9, and actin cytoskeleton and endocytosis related upregulated proteins, like Pstpip1 and Ppp1r9b. The proteomic results in this study shed light on the mechanism of antiphagocytosis and innate immunity of macrophages infected with △*MetQ* and ZY05719, which might provide novel targets to prevent or control the infection of SS2.

## Introduction

*Streptococcus suis* (*S. suis*) is an important zoonotic pathogen that can cause severe diseases in human and pigs, including streptococcal toxic shock syndrome (STSS), meningitis and septicemia; it has led to great threat to public health and has resulted in huge economic losses. Based on the antigenicity of capsular polysaccharide of *Streptococcus suis*, 29 serotypes of *Streptococcus suis* have been identified (Gottschalk et al. 2010). And serotype 2 *Streptococcus suis* is the most prevalent serotype isolated in most countries (Fittipaldi et al. 2012). *Streptococcus sui*s ZY05719 is a serotype 2 strain isolated from an outbreak in China. Since *S. suis* has caused STSS and acute death in a clinical setting, we proposed that the *S. suis* is able to evade the innate immune response of the host. Phagocytosis is an important process in innate immunity. Although numerous virulence factors have been previously reported, the pathogenesis of *S. suis* still needs to be elucidated. From our previous research, a *MetQ* transposon mutant strain showed decreased anti-phagocytosis ability. Anti-phagocytosis is important for the immune evasion of pathogens. Hence, the mechanism of *MetQ* associated anti-phagocytosis is of interest for further exploration.

MetQ is a putative methionine ABC transporter substrate-binding protein encoded by *MetQ* gene, which is involved with uptake of environmental methionine (Hullo et al. 2004; Nguyen et al. 2015). *MetQ* has been shown to contribute to the survival of *Neisseria gonorrhoeae* in macrophages and its adhesion to epithelial cells (Semchenko et al. 2017). Expression of *MetQ* increased upon infection of *S. suis*, indicating *MetQ* was a potential virulence factor involved with in vivo infection (Gu et al. 2009). Methionine is an amino acid required for the initiation of protein biosynthesis. Methionine is also associated with the biosynthesis of S-adenosylmethionine, which contributes to the methylation of DNA, RNA and proteins (Husna et al. 2018; Parkhitko et al. 2019). Prokaryotes have the capability of de novo methionine biosynthesis, however, vertebrates do not possess the methionine biosynthesis pathways (Husna et al. 2018).

Pathogens could invade the host and trigger the immune responses. Macrophages are important for innate immunity by ingestion and elimination of microorganisms, and they also present microbial antigens on their cell surfaces (Flannagan et al. 2009). In turn, pathogens have evolved to fight against macrophages by inhibiting the phagocytosis receptors or by hijacking the pathways involved with the actin cytoskeleton (Shao et al. 2002; Caron et al. 2006; Carlin et al. 2009). In addition to the phagocytosis function, macrophages could also secrete cytokines and chemokines and play an important role in inflammation. Cytokines are beneficial to the host when secreted at appropriate amounts, but they can cause damage when overproduced (Laskin 2009). Chemokines help with recruitment of immune cells to sites of inflammation (Comerford and McColl 2011). Pathogen infection normally elicits an inflammatory response, and in turn, some pathogens may interrupt cytokine secretion to impair the immune response, or even take advantage of the immune response (Sibley 2011; Hall and Simmonds 2014).

Since △*MetQ* showed decreased anti-phagocytosis ability, we were interested in the mechanism of its role in anti-phagocytosis and pathogenesis. To explore the differential protein expression of RAW264.7 cells upon infection with ZY05719 and △*MetQ*, we conducted a comparative proteomics assay based on stable isotope labeling of amino acids in cell culture (SILAC) coupled with liquid chromatography–tandem mass spectroscopy (LC–MS). The quantitative proteomics results showed 70 upregulated proteins and 40 downregulated proteins in △*MetQ* infected cells compared with infected wild-type cells. The quantitative data and bioinformatic analysis might help further characterize these immune related proteins and their role in pathogenesis of *S. suis*.

## Materials and methods

### Bacteria and cells

*Streptococcus suis* ZY05719 was obtained from a diseased pig in an outbreak in Ziyang, China. (The genome of Streptococcus suis ZY05719 has been sequenced and uploaded to NCBI with "Accession: NZ_CP007497.1 GI: 820722437". This strain has been published a number of articles as a model strain. The strain ZY05719 is deposited in OIE Reference Lab for Swine Streptococcosis.) △*MetQ* was obtained by deletion of the *MetQ* gene (ZY05719_08420) of ZY05719 and was conserved in this lab. The bacteria were cultured in Todd Hewitt Broth (THB) (BD Biosciences, San Jose, CA, USA) at 37 °C.

RAW264.7 cell was purchased from ATCC. SILAC labeled RAW264.7 cells was stored in this lab. Briefly, the cells were labeled by heavy isotope (Arg^13^C_6_, Lys^13^C_6_) and light isotope (Arg^12^C_6_, Lys^12^C_6_) respectively in SILAC Dulbecco’s modified Eagle medium (DMEM) supplemented with 10% FBS (fetal bovine serum; Pierce, Rockford, IL, USA) at 37 °C under 5% CO_2_. For SILAC labeling, the cells were passaged for 5 generations.

### Sample preparation

ZY05719 and △*MetQ* were cultured overnight on THB agar at 37 °C. A colony was picked and inoculated into 5 mL of THB at 37 °C to an OD_600_ of 0.8. The bacteria were centrifuged at 5000*g* for 5 min and washed 3 times with phosphate buffered saline (PBS). The ZY05719 were then resuspended with heavy isotope DMEM and △*MetQ* with light isotope DMEM. The heavy isotope-labeled RAW264.7 cells were infected with ZY05719 and light-labeled RAW264.7 cells were infected with △*MetQ* at a multiplicity of infection (MOI) of 10:1 and incubated for 4 h at 37 °C under 5% CO_2_. After incubation, the total protein of RAW264.7 cells was extracted with RIPA Lysis and Extraction Buffer supplemented with Protease Inhibitor Cocktail (Thermo, Waltham, MA, USA). Protein concentrations were measured by BAC Protein Assay Kit (Pierce, Rockford, IL, USA). Equal amount of protein from each lysate was mixed in a new tube. Mixed lysates were subjected to SDS-PAGE and each gel was excised into five slices according to the abundance. Two biological replicates were performed for the experiment.

### Mass spectrometry analysis

The gels were decolorized by 200 μL of 100 mM ammonium bicarbonate/30% acetonitrile (ACN) and washed to transparency state. The supernatant was discarded and 10 mM DTT (dithiothreitol) was added and incubated for 1.5 h at 37 °C. The supernatant was removed and 100% ACN was added, which was discarded after 5 min. Then 60 mM iodoacetamide (IAA) was added and the treatment was performed in the dark for 20 min. The liquid was discarded, and 100 mM ammonium bicarbonate was added for a 15 min reaction. The supernatant was removed and 100% ACN was added and incubated for 5 min before discarding. After thorough drying, the lyophilized gel was digested with 15 μL of 10 ng/μL trypsin (Promega) at 37 °C overnight. The tryptic digests were separated with Easy-nLC 1000 System (Thermo, Waltham, MA, USA). After the peptides were separated by chromatography, mass spectrometry analysis was conducted by a Q-Exactive mass spectrometer (Thermo Scientific). Scanning range of precursor ion was 300–1800 m/z. The resolution of full mass spectrometry was 70,000 fwhm (full width at half maxima) and the full scan AGC contained 3 × 10^6^ targets. The Maximum IT was 50 ms. The MS2 had a resolution of 17,500 fwhm, maximum IT of 60 ms, normalized collision energy of 27 eV and dynamic exclusion of 60.0 s. The activation type of MS2 was HCD.

### Data analysis and bioinformatics analysis

The raw MS/MS data was searched against UniProt *Mus musculus* database and analyzed by MaxQuant software for protein identification and quantification. A 1.5-fold change of protein abundance ratio and a *p*-value ≤ 0.05 was chosen to indicate significant change, based on a previous research in this lab (Jie et al. 2017). Differentially expressed proteins were analyzed with Gene Ontology (GO) and Kyoto Encyclopedia of Genes and Genomes (KEGG) analysis. The upregulated and downregulated proteins were analyzed separately. GO classifications were analyzed by Blast2Go software and categorized into biological process (BF), molecular function (MF) and cellular component (CC). KEGG classification was conducted with KAAS (KEGG Automatic Annotation Server) software to explore the involvement of signal pathways in differentially abundant proteins. The enrichment analysis was performed by KOBAS software. Protein–protein interaction network was analyzed by STRING and Cytoscape software.

### Validation of proteomics results by quantitative real-time polymerase chain reaction and Western Blot

ZY05719 and △*MetQ* were cultured to an OD_600_ of 0.6–0.8, washed 3 times with PBS and resuspended with DMEM without FBS. RAW264.7 cells were infected with the two respective strains at a MOI of 10:1 for 4 h. Total RNA was extracted with the RNAiso Plus (Takara, Tokyo, Japan) according to the manufacturer’s instructions. cDNA was synthesized with PrimeScript RT reagent kit (TaKaRa) as per the protocol. mRNA levels were examined using a SYBR Premix Ex TaqTM kit (TaKaRa) in an ABI Step One Plus Real-Time PCR System. Seven genes that are immune system and signal transduction related were selected for examination. Primers are listed in Table 1, and GAPDH was used as a reference gene. Relative expression levels of target genes were calculated using the 2^−△△CT^ method.

**Table 1:** Primers used for RT-qPCR

Name	Oligonucleotide sequence (5′-3′)
Spp1-f	ATCTCACCATTCGGATGAGTCT
Spp1-r	TGTAGGGACGATTGGAGTGAAA
src-f	GAACCCGAGAGGGACCTTC
src-r	GAGGCAGTAGGCACCTTTTGT
Clec4e-f	GCTCTCCTGGACGATAGCC
Clec4e-r	TGCGATATGTTACGACACATCTG
cebpd-f	CGACTTCAGCGCCTACATTGA
cebpd-r	CTAGCGACAGACCCCACAC
Mcl1-f	AAAGGCGGCTGCATAAGTC
Mcl1-r	TGGCGGTATAGGTCGTCCTC
ccl9-f	CCCTCTCCTTCCTCATTCTTACA
ccl9-r	AGTCTTGAAAGCCCATGTGAAA
Rab5a-f	GCTAATCGAGGAGCAACAAGAC
Rab5a-r	CCAGGCTTGATTTGCCAACAG
GAPDH-F	CGTGTTCCTACCCCCAATGT
GAPDH-R	TGTCATCATACTTGGCAGGTTTCT

Src, Pstpip1 and Hmox1 were chosen for Western Blotting confirmation of the high-throughput results. In brief, total protein were extracted from the two strains after 4-h infection, respectively. Proteins were separated by SDS-PAGE and then transferred to 0.22 μm polyvinylidene difluoride (PVDF) membrane and blocked with 5% skim milk (BD Biosciences). Membranes were incubated with primary antibodies Src (Proteintech), Pstpip1 (Proteintech), Hmox1 (Abcam) and GAPDH (CMCTAG) overnight at 4 °C. After incubation, membranes were washed 3 times with TBST, and incubated with secondary antibodies HRP Goat anti-Mouse or Rabbit IgG antibodies (CMCTAG). Proteins of interest were detected with ECL Pico-Detection Western Blotting Substrate (CMCTAG).

### Phagocytosis assay and intracellular survival assay

A phagocytosis assay was performed according to previous research with a few modifications (Segura et al. 2004). ZY05719 and △*MetQ* were grown to an OD_600_ of 0.6–0.8, washed three times with PBS and resuspended with DMEM. RAW264.7 cells were infected separately with both strains at a MOI of 10:1. After 1 h infection, the cells were washed three times with PBS and incubated with DMEM supplemented with 100 μg/mL gentamicin and 10 μg/mL penicillin G for 1 h to eliminate extracellular bacteria. The cells were then washed three times with PBS for 3 times, and lysed in 1 mL ddH_2_O. Phagocytosed bacteria were diluted and plated on THA for counting. The intracellular survival assay was conducted as described previously (Tang et al. 2012). After the bacteria were phagocytosed by macrophages, the cells were incubated with antibiotics, and lysed with 1 mL ddH_2_O at different time points of 1 h and 2 h. The intracellular survival rates were calculated as CFU_2 h_/CFU_1 h_ × 100%.

### Whole blood survival assay

The whole blood survival assay was conducted based on a previous report with slight modifications (Yamaguchi et al. 2006). ZY05719 and △*MetQ* were grown to an OD_600_ of 0.6–0.8 in THB, washed three times with PBS and resuspended in PBS. Blood samples were obtained from healthy pigs. Bacteria were diluted to an OD_600_ of 0.1 in PBS. 100 μL of bacteria were added to 1 mL of pig blood, and incubated for 3 h at 37 °C. Bacteria were diluted and plated on THB agar at 0 h and 3 h. The survival rates were calculated as CFU_3 h_/CFU_0 h_ × 100%.

### Statistical analysis

Data were analyzed using unpaired unpaired two-tailed Student’s *t*-test with GraphPad Prism 5 software. Data were expressed as the means with standard error of the mean (SEM). A *p* value < 0.05 was considered statistically significant.

## Results

### Anti-phagocytosis ability, intracellular survival ability and whole blood survival rate decreased in △*MetQ*

**Fig. 1:**
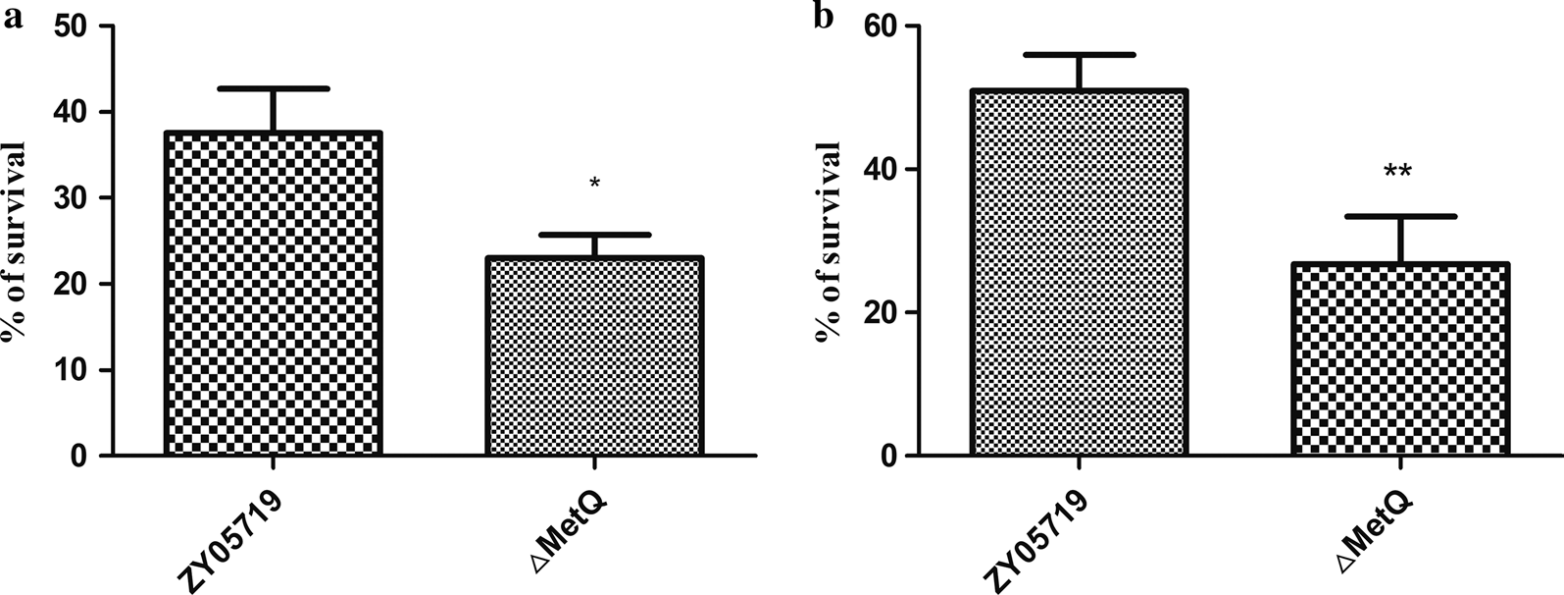
Antiphagocytosis ability, intracellular survival ability and whole blood survival ability of △*MetQ*. **a** Intracellular survival rate of △*MetQ* and ZY05719 in RAW264.7 cells. **b** Survival rate of △*MetQ* and ZY05719 in whole pig blood. The statistical significance was determined by Student’s *t* test. *p < 0.05; **p < 0.01; ***p < 0.001

We have found that △*MetQ* had decreased anti-phagocytic ability compared to ZY05719 (Pei et al. 2020), and was more easily killed by macrophage RAW264.7 cells when phagocytosed (*p* < 0.05, Fig. 1a). In the whole blood survival assay, △*MetQ* showed lower viability than wild-type ZY05719 (*p* < 0.05, Fig. 1b). Therefore, in this experiment, we chose the macrophage RAW264.7 cell line for further research to identify alterations of immune related proteins.

### Protein identification and quantification

More than 3000 proteins were identified by LC–MS analysis in this assay. Between the ZY05719 and △*MetQ* infected group, there were 110 proteins that showed differential abundances, in which 70 proteins were upregulated with △*MetQ* infected macrophages compared with wild-type infected cells (light/ heavy ratio ≥ 1.5, *p* ≤ 0.05) and 40 proteins were downregulated (light/heavy ratio ≤ 0.67, *p* ≤ 0.05). All proteins detected by LC–MS analysis are listed in Additional file 1: Table S1. All differentially abundant proteins are listed in Additional file 2: Table S2. Cells were labeled previously in this lab (Jie et al. 2017). The mass spectrometry proteomics data have been deposited to the ProteomeXchange Consortium via the PRIDE partner repository with the dataset identifier PXD018091.

### Bioinformatics analysis

Since macrophages are involved in immune related process such as phagocytosis and inflammatory reactions, and the two strains showed significant resistances to phagocytosis, we paid more attention on the immune response associated proteins.

**Fig. 2:**
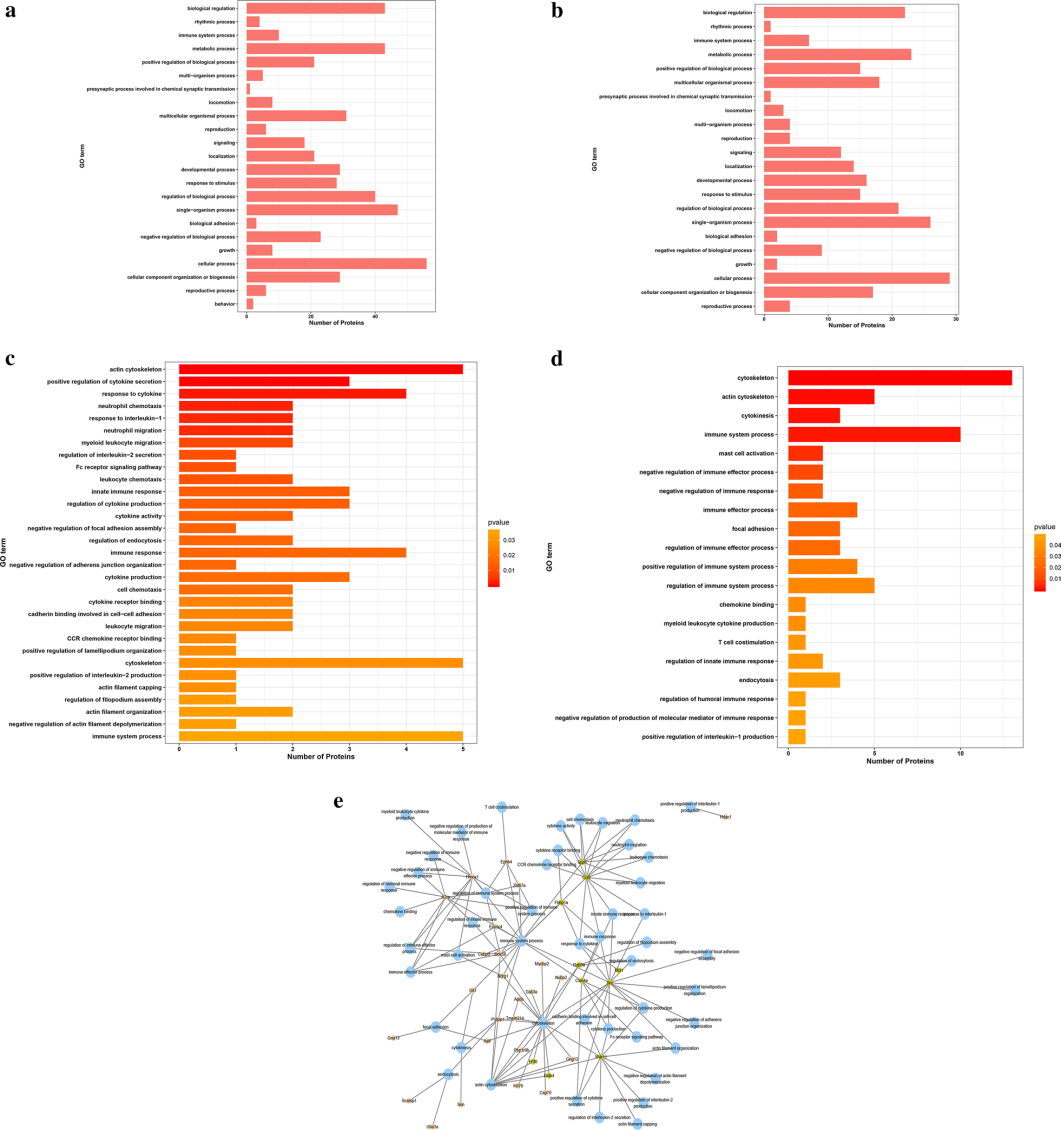
GO analysis of differentially expressed proteins. **a** Upregulated proteins were categorized into the level 2 biological process GO terms. **b** Downregulated proteins were classified into level 2 biological process GO terms. **c** Immunity related downregulated proteins from the GO enrichment analysis. **d** Immunity related upregulated proteins from the GO enrichment analysis. **e** Connections of the upregulated and downregulated immunity related proteins. Green circle indicated the downregulated protein, orange circle indicated upregulated protein, blue circle represented the GO terms

The GO database is classified into three categories: biology process, molecular function and cellular components. Proteins of increased and decreased abundances were analyzed for GO category enrichment. The immune system process from biology process included 10 increased abundance and 7 decreased abundance immune system process proteins (Fig. 2a, b, Table 2). Through GO Enrichment analysis, we focused on some immunity related processes. Immunity related GO Enrichment results are shown in Fig. 2c (decreased abundance proteins) and Fig. 2d (increased abundance proteins). Network (Fig. 2e) showed the connections of the immune related GO terms and their related genes.

**Table 2:** Immune system process related proteins through GO annotation

	Protein	Gene
Upregulated	CCAAT/enhancer-binding protein delta	Cebpd
	Proline–serine–threonine phosphatase-interacting protein 1	Pstpip1
	Heme oxygenase 1	Hmox1
	Alpha-2-macroglobulin; Alpha-2-macroglobulin 165 kDa subunit; Alpha-2-macroglobulin 35 kDa subunit	A2m
	Probable ATP-dependent RNA helicase DDX58	Ddx58
	Exosome complex component RRP41	Exosc4
	Transcription factor SOX-14	Sox14
	Zinc finger and BTB domain-containing protein 7A	Zbtb7a
	Ephrin type-B receptor 4	Ephb4
	Protein NDRG1	Ndrg1
Downregulated	Peptidyl-prolyl cis–trans isomerase FKBP1A; Peptidyl-prolyl cis–trans isomerase	Fkbp1a
	Induced myeloid leukemia cell differentiation protein Mcl-1 homolog	Mcl1
	C–C motif chemokine 9; CCL9(29–101);CCL9(30–101);CCL9(31–101)	Ccl9
	Neuronal proto-oncogene tyrosine-protein kinase Src; Tyrosine-protein kinase Yes; Tyrosine-protein kinase Fyn; Proto-oncogene tyrosine-protein kinase LCK	Src;Yes1; Fyn;Lck
	Spectrin beta chain, brain 1	Sptbn1; Spnb2
	C-type lectin domain family 4 member E	Clec4e
	Osteopontin	Spp1

**Fig. 3:**
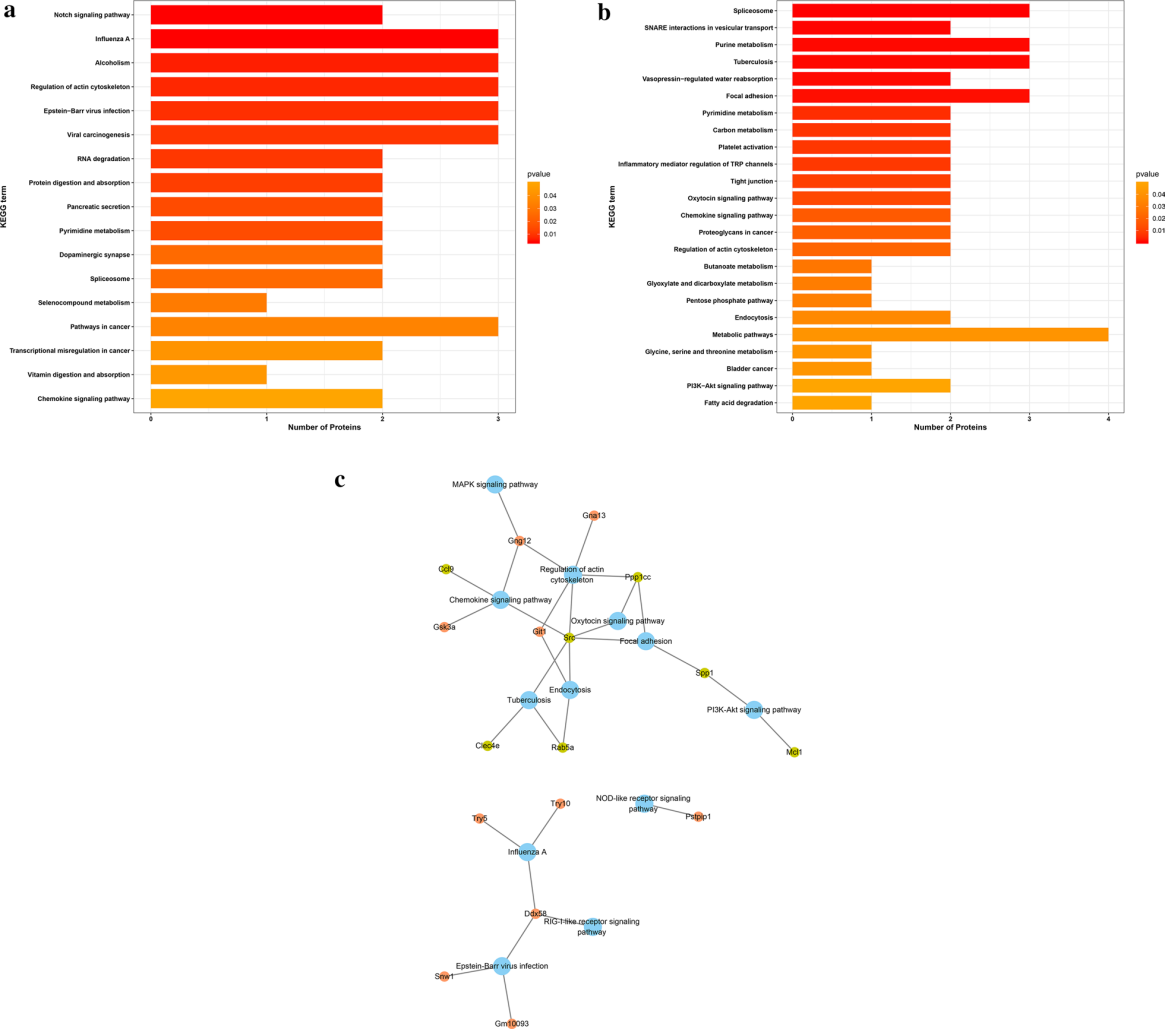
KEGG pathway analysis. **a** KEGG enrichment analysis of upregulated proteins. **b** KEGG enrichment analysis of downregulated proteins. **c** Network of immunity related KEGG pathways

The KEGG analysis were performed to determine the relationships between differently expressed proteins and their related signaling pathways. KEGG enrichment analysis results were conducted separately for proteins of increased and decreased abundances as presented in Fig. 3a, b. We have found the pathways included many aspects of cellular functions, and especially the metabolism process. In the KEGG pathway results for proteins of decreased abundance, pathways involved in tuberculosis, focal adhesion, tight junction, regulation of actin cytoskeleton, chemokine signaling and endocytosis were noted for their immune related functions. In the KEGG pathway results for proteins of increased abundance, influenza A, regulation of actin cytoskeleton, Epstein–Barr virus infection and chemokine signaling pathways attracted our interest. The connections of the genes involved with the pathway are depicted in a network (Fig. 3c).

**Fig. 4:**
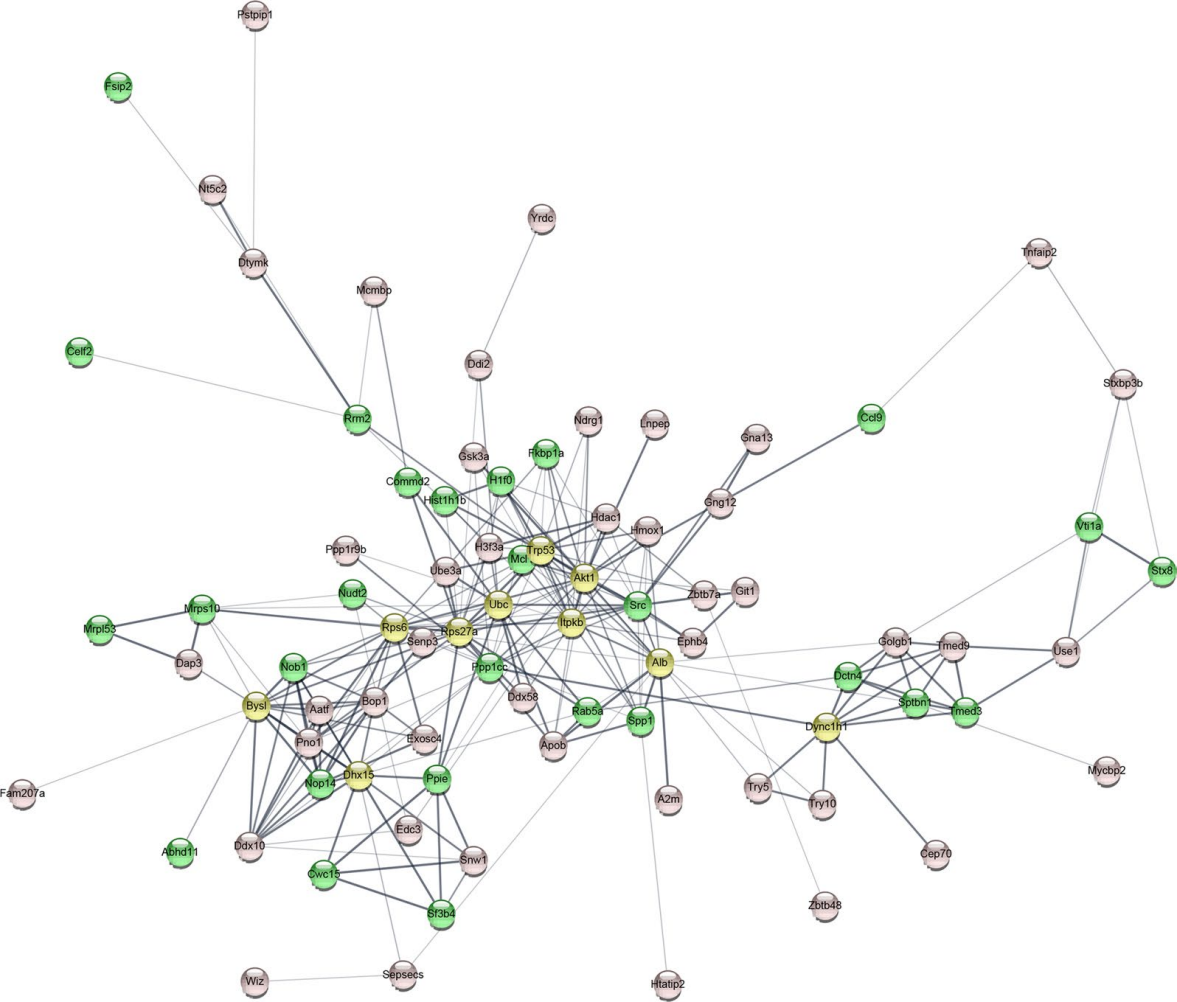
The protein–protein interaction network of the differentially expressed proteins were analysed by STRING and presented with Cytoscape software. Pink bubbles represented upregulated proteins, green bubbles indicated downregulated proteins, yellow ones were related proteins while not identified as differential abundance proteins

Possible protein–protein interactions among the differentially abundant proteins were analyzed with STRING software, and are presented using Cytoscape software (Fig. 4). Upregulated proteins are denoted with pink bubbles, whereas downregulated proteins are in green bubbles; yellow bubbles represent proteins which were not differentially abundant by LC–MS.

### Validation of protein expression by qPCR and Western blotting

**Fig. 5:**
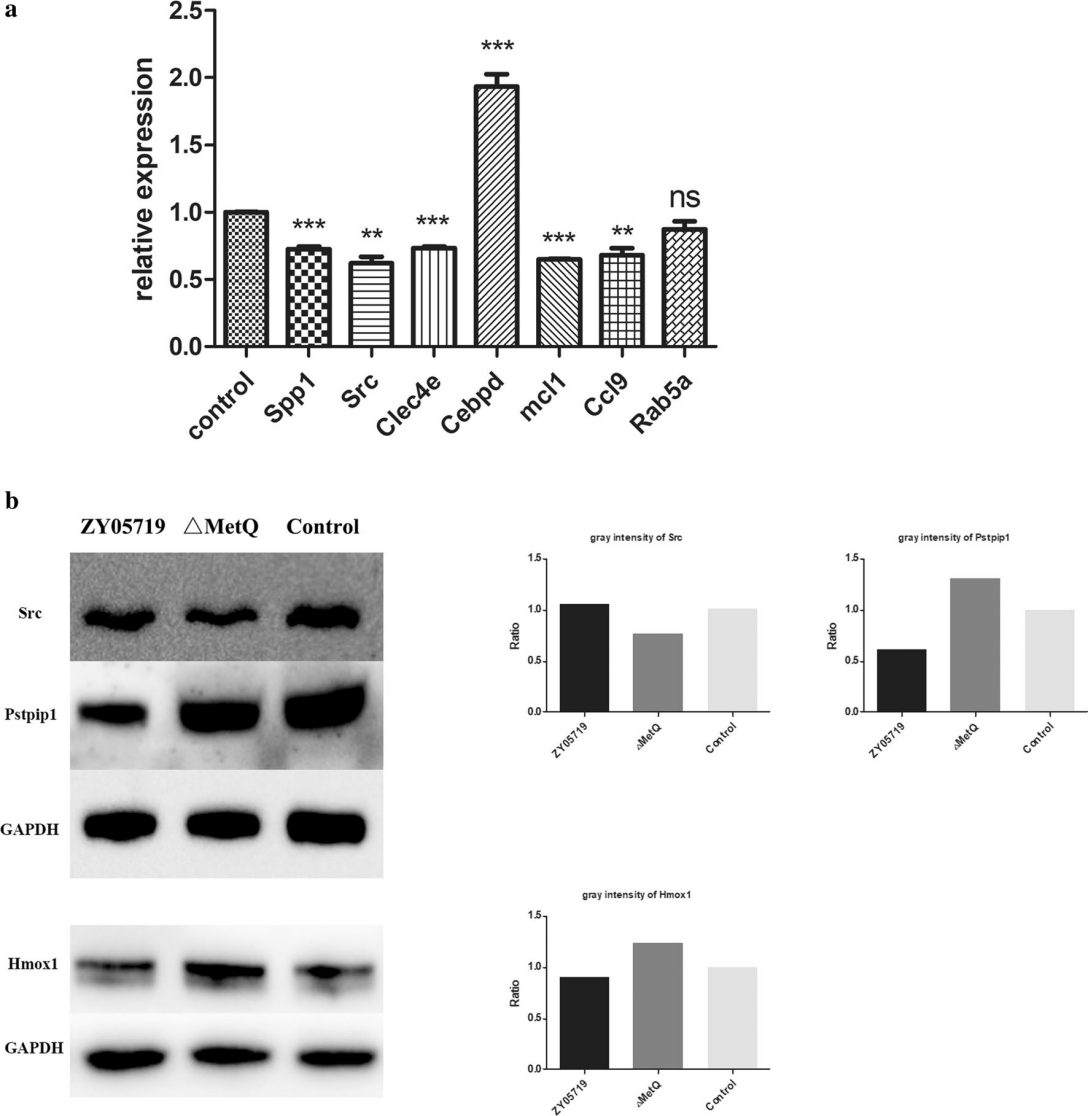
Validation of proteomics results. **a** Seven genes encoding for the corresponding differentially expressed proteins were selected to verify the proteomics results by qPCR. The results were calculated by 2^−△△CT^ method. **b** Pstpip1, Src and Hmox1 proteins were selected for the validation of the quantitative proteomics. GAPDH was used as reference. The gray intensities are shown on the right. ZY05719, ZY05719 treated cells; △*MetQ*, △*MetQ* treated cells; control, non-infected cells. The statistical significance was determined by Student’s *t* test. *p < 0.05; **p < 0.01; ***p < 0.001

We selected 7 genes that showed differential abundances by LC–MS data for qPCR verification. RAW264.7 cells were infected with ZY05719 and △*MetQ* for 4 h separately and transcription of selected genes were examined. qRT-PCR showed transcriptional downregulation of *Spp1*, *Src*, *Clec4e*, *Mcl1* and *Ccl9* genes, and upregulation of *Cebpd* in correlation with protein abundance level determined by LC–MS, though some did not reach the twofold change criteria (Fig. 5a).

Western blotting assays were performed to detect the different levels of Pstpip1, Src and Hmox1. It was shown that after infection with ZY05719 and △*MetQ* respectively, abundances of Pstpip1 and Hmox1 increased, while Src abundance was reduced in the △*MetQ* treated group, which was in agreement with the LC–MS results (Fig. 5b).

## Discussion

*Streptococcus suis* serotype 2 (SS2) is an important zoonotic pathogen that causes acute sepsis, meningitis, and streptococcal toxic shock syndrome (STSS), resulting in severe threats to humans and losses to the swine industry. SS2 can cause acute death, which led us to postulate that SS2 may evade the innate immunity. In *Streptococcus pneumoniae*, MetQ was responsible for methionine uptake, while MetE and MetF were required for methionine biosynthesis (Basavanna et al. 2013). However, methionine acquisition is not totally reliant upon these *Streptococcus pneumonia* genes. There may be a second methionine uptake system similar to the BcaP system of *Lactococcus lactis* to obtain methionine from the environment (den Hengst et al. 2006; Basavanna et al. 2013). Methionine synthesis and transport genes were reported to be related to the virulence of *Brucella melitensis*, *Salmonella enterica* and *Streptococcus suis* (Lestrate et al. 2000; Ejim et al. 2004; Arenas et al. 2019).

Phagocytosis is an essential step for innate immunity. Hence, the anti-phagocytosis ability of SS2 attracted our interest. From our previous research, we identified *MetQ* was involved with phagocytosis resistance of SS2 (Pei et al. 2020). By deletion of *MetQ* (△*MetQ*), we discovered the △*MetQ* strain showed attenuated intracellular survival and whole blood survival. In this study, we used SILAC based LC–MS technology to explore differentially abundant proteins when macrophage RAW264.7 cells were infected with wild-type strain ZY05719 and △*MetQ*. We aimed to explore differentially expressed phagocytosis and immunity related proteins to elucidate the pathogenesis of SS2.

From LC–MS results, we have identified 110 proteins of differential abundance (70 increased abundance and 40 decreased abundance proteins). GO annotation results showed 10 increased abundance proteins and 7 decreased abundance proteins were associated with immune system process. GO over-representation analysis showed more immunity GO terms, including endocytosis, actin cytoskeleton, cytokine production and chemotaxis related GO terms. KEGG analysis was separately conducted for increased and decreased abundance proteins. Among the increased abundance proteins, enriched KEGG pathways included regulation of actin cytoskeleton and chemokine signaling pathway which were directly related to the processes of endocytosis and immunity. While among the decreased abundance proteins, more KEGG pathways were associated with immune system; for example, focal adhesion, chemokine signaling pathway, regulation of cytoskeleton and endocytosis pathways.

Phagocytosis is a fundamental process in engulfment and elimination of pathogens. Cells migrate to a certain site when sensing the chemoattractants, which is known as chemotaxis; this is another important immune function of macrophages. Phagocytosis and chemotaxis involve the recruitment of actin and remodeling of the actin cytoskeleton. Rho family GTPases are important regulators for the actin recruitment (Mao and Finnemann 2015). It was also reported that focal adhesion might be associated with the phagocytosis process since focal adhesion proteins, talin, paxillin, vinculin and focal adhesion kinase (FAK) were enriched in FcγR and CR3 phagosomes (May and Machesky 2001). Production of pro-inflammatory cytokines and chemokines are also important functions of phagocytes (Moon et al. 2011; Xuan et al. 2015).

Actin cytoskeleton is crucial for the phagocytosis process. The actin remodeling is regulated by the Rho GTPase family. Among the Rho GTPases, RhoA, Rac1, and Cdc42 are best studied. And for some pathogens, they have evolved to counteract the phagocytosis and immune responses of the host by hijacking the actin cytoskeleton related proteins. For example, *Yersinia* YopO could mimic the function of host GDI, thus inhibiting activation of RhoA and Rac 1 (Prehna et al. 2006). YopE of *Y. enterocolitis* could inhibit RhoG which led to the phagocytosis resistance of the bacteria (Roppenser et al. 2009). For SS2, however, capsule of SS2 is the best studied antiphagocytic factor. The mechanism of the phagocytosis resistance of SS2 needs further research.

Through GO and KEGG analysis, we have identified a multifunctional protein, Src, which had reduced abundance in the △*MetQ* treated group, and its expression was verified to be downregulated by Western blotting assay. We have also discovered that Src is involved with many immune related processes, such as actin organization and endocytosis functions. Src family tyrosine kinases were reported to contribute to the phagocytosis and cytokine activation after *Borrelia burgdorferi* infection (Killpack et al. 2017). Src family kinases were responsible for signal transduction in immune cells, acting as immunoreceptors, cytokine receptors and integrin receptors. To complicate matters, Src family kinases are involved with regulating both activation and inhibition pathways in the immune cells (Lowell 2011). Therefore, the relationship between Src and the phagocytosis phenotype in our experiments was hard to determine.

Pstpip1 is a F-BAR domain containing protein that is involved with PAPA syndrome. Pstpip1 can bind to Wiskott-Aldrich syndrome protein (WASP) and regulate the actin cytoskeleton, and hence, modify podosomes and filopodia formation in macrophages (Starnes et al. 2014). Pstpip1 has been shown to interact with pyrin, and hence, activate caspase-1. Activated caspase-1 can in turn produce cytokines IL-1β and IL-18, and lead to inflammation (Shoham et al. 2003; Yu et al. 2007). Pstpip1 in T cells could connect the CD2 molecule and WASP and therefore contribute to the formation of immunological synapses (Badour et al. 2003).

Ppp1r9b (Neurabin2) is a scaffold protein that can bind F-actin to regulate the actin cytoskeleton organization (Satoh et al. 1998). Git1 is an ARF GTPase-activating protein which binds ARFs, PIX and FAK proteins, and it regulates adhesion and cytoskeleton organization (Turner et al. 2001). Gna13 (Guanine nucleotide-binding protein subunit alpha-13) can activate RhoA, which is crucial for actin cytoskeleton organization endocytosis process (Kreutz et al. 2006). SCAMP1 (Secretory carrier membrane proteins) can bind to intersecin1, which facilitates the formation of clathrin-coated vesicles (Hussain et al. 2001; Almeida-Souza et al. 2018). Hmox1 (Heme oxygenase-1) is an anti-inflammatory factor in the microbial infection process. Hmox1 facilitates the phagocytosis of zymosan A, which is initiated by Taurine chloramine (Poss and Tonegawa 1997; Kim et al. 2018). Dctn4 (dynactin subunit 4) is a component of dynactin which can bind the Arp1 subunit of dynactin, and is related to dynein-mediated vesicle motility (Karki et al. 2000). Rab family small GTPases are multifunctional, and their roles vary from regulation of cell proliferation, signal transduction to endocytosis and so forth. Rab5a localizes to early endosomes and regulates receptor-mediated endocytosis, actin cytoskeleton and T cell migration through Rho GTPase family protein Rac (Ong et al. 2014). *Leishmania donovani *infection has been shown to upregulate Rab5a expression in human macrophages, thereby blocking lysosome fusion with early endosomes and facilitating parasite survival in human phagocytes (Verma et al., 2017). Our study showed downregulation of *Rab5a* expression in the △*MetQ* treated group, and Rab5a downregulation in △*MetQ* treated group might contribute to the attenuated survival of △*MetQ* in macrophages. EGF was shown to stimulate endocytosis, and relies on Rab5a activation (Barbieri et al. 2000). Classical swine fever virus (CSFV) endocytosis by macrophages requires Rab5 (Zhang et al. 2018). Entry of Japanese encephalitis virus (JEV) into BHK-21 cells was Rab5-dependent (Liu et al. 2017). From our analysis, we discovered upregulated and downregulated endocytosis and actin related proteins, indicating a complicated network in the regulation of this process. To determine the function of these proteins, further research is needed.

In conclusion, we conducted the comparative proteomics assay on infection of RAW264.7 cells with △*MetQ* and ZY05719. Differentially expressed proteins were analyzed with bioinformatics tools, and were found to be involved with actin cytoskeleton, neutrophil chemotaxis, cytokine responses and immune responses; these results underlie the complicated regulation of the host cell pathways. A greater focus was placed on upregulated actin cytoskeleton related proteins, which may contribute to the antiphagocytosis phenotype, and also, the immune response related proteins that may contribute to the attenuated pathogenesis of △*MetQ.* The proteins with significant alterations in abundance together with the bioinformatics analysis provides insight on the mechanism of SS2 antiphagocytosis. These proteins of interest will be further explored for their functions on SS2 in future experiments.

## Supplementary information

**Additional file 1: Table S1.** All proteins identified by LC–MS.

**Additional file 2: Table S2.** All differentially expressed proteins.

## Data Availability

All datasets supporting the conclusion of this article are included within the article and its additional files.
